# Multi factorial interactions in the pathogenesis pathway of Alzheimer’s disease: a new risk charts for prevention of dementia

**DOI:** 10.1186/1742-4933-7-S1-S4

**Published:** 2010-12-16

**Authors:** Federico Licastro, Elisa Porcellini, Paola Forti, Massimo Buscema, Ilaria Carbone, Giovanni Ravaglia, Enzo Grossi

**Affiliations:** 1 Department of Experimental Pathology, Via San Giacomo 14, 40126, Bologna, Italy; 2 Department of Internal Medicine, Cardioangiology and Hepatology, School of Medicine, Via Albertoni 15, 40138, Bologna, Italy; 3 Semeion Research Center of Communication Science, Via Sersale 117, 00128 Roma, Italy; 4 Centro Diagnostico Italiano, Milano, Italy

## Abstract

**Background:**

The population longitudinal study named “The Conselice Study” has been the focus of the present investigation. 65 years old or older participants of this population study on brain aging were followed up for 5 years: 937 subjects completed the follow-up. Relationships of 46 genetic, phenotypic, clinical and nutritional factors on incident cognitive decline and incident dementia cases were investigated.

**Results:**

A new statistical approach, called the Auto Contractive Map (AutoCM) was applied to find relationship between variables and a possible hierarchy in the relevance of each variable with incident dementia. This method, based on an artificial adaptive system, was able to define the association strength of each variable with all the others. Moreover, few variables resulted to be aggregation points in the variable connectivity map related to cognitive decline and dementia. Gene variants and cognate phenotypic variables showed differential degrees of relevance to brain aging and dementia. A risk map for age associated cognitive decline and dementia has been constructed and will be presented and discussed.

**Conclusion:**

This map of variables may be use to identify subjects with increased risk of developing cognitive decline end/or dementia and provide pivotal information for early intervention protocols for prevention of dementia.

## Background

### Inflammatory responses during ageing

A dramatic increase in mean life span and life expectancy, coupled with a significant reduction in early mortality, has lead to a substantial increment in the number of elderly population in contemporary societies. This demographic picture parallels the merging of a new epidemic characterized by chronic age related diseases. Most age related diseases have complex aetiology and pathogenesis. Clinical diagnosis and therapy of these diseases imply multidisciplinary medical approaches and their cost is progressively increasing.

The immune system is often implicated, with a variable degree of importance, in almost all age related diseases or associated with their clinical complications. Both innate and clonotypic immune system are usually involved in the pathogenesis of these chronic diseases. However, inflammatory responses appear to be the prevalent trigger mechanism driving tissue damages associated with different age-related [1].

Chronic inflammation is involved in the pathogenesis of all age-related diseases: Alzheimer's disease, atherosclerosis, diabetes, autoimmune diseases, sarcopenia and cancer have an important inflammatory component. Furthermore, increased levels of circulating inflammatory mediators may result from a constant, low-grade activation of cytokine producing cells or a dysregulated cytokine response following stimulation [2].

However, recent researches link an individual’s exposure to precedent infections which have become latent infections and are able to induce chronic inflammation. A continuous chronic activation of immune responses then may lead and to increased risk of heart attack, stroke, and cancer. For example, the risk of heart attack and stroke is correlated with serum levels of inflammatory proteins such as CRP. Within individuals, CRP levels are also correlated to the number of seropositivities to common pathogens, indicating a history of infections (FINCH).

Low-grade increment of circulating TNF-α, IL-6, soluble IL-2 receptor (sIL-2R), and C reactive protein (CRP) and decreased levels of albumin and cholesterol, which also are indicators of inflammatory state, are strong predictors of all-cause mortality risk in longitudinal studies of several elderly cohorts. The effects of inflammatory mediators are independent of pre-existing morbidity and of other traditional risk factors for death (smoking, blood pressure, physical exercise, total cholesterol, co-morbidity, body mass index, and intake of anti-inflammatory drugs) in survival analyses suggesting that cytokines trigger/exaggerate pathological processes or act as very sensitive markers of subclinical disorders in elderly populations [2-8].

Therefore, innate immunity appears to play a pivotal role in several age related diseases and therapeutic control of chronic inflammation is becoming an emerging topics of modern gerontology and clinical geriatrics.

### Brain degenerative diseases: Alzheimer’s disease

Alzheimer’s disease (AD) is a heterogeneous and progressive neurodegenerative disease that in Western societies accounts for the majority of clinical senile dementia and by 2050 the number of patients with AD is expected to rise from 4.6 to 16 millions cases in the USA [9]; worldwide statistical projections predict more that 45 million of AD patients within the above year. Neuropathological hallmarks of AD are extracellular amyloid deposits (neuritic plaques) and intracellular deposition of degenerate filaments (neurofibrillary tangles) [10]. Major clinical manifestations of the disease are memory loss and cognitive impairment [11].

Inflammation clearly occurs in pathologically vulnerable regions of the AD brain, and it does so with the full complexity of local peripheral inflammatory responses. In the periphery, degenerating tissue and the deposition of highly insoluble abnormal materials are classical stimulants of inflammation. Likewise, in the AD brain damaged neurons and neurites and highly insoluble Aβ42 peptide deposits and neurofibrillary tangles provide obvious stimuli for inflammation. Senile plaques in AD brains are associated with reactive astrocytes and activated microglial cells and cytokines and acute phase proteins are overexpressed in microglia and astrocytes surrounding neuropathological lesions in AD brains. Inflammatory factors, such as cytokines, chemokines, complement components and acute phase proteins co-localize as secondary components in neuritic or senile plaques or are over-produced in AD brains, and activated microglia surround senile plaques and areas of neurodegeneration [12,13]. There is accumulating evidence that Aβ peptide may promote or exacerbate inflammation by inducing glial cells to release immune mediators. Moreover, microglial and astroglial cells surrounding mature plaques in AD brains have been found to express activation markers. Enriched populations of human microglial cells isolated from mixed cell cultures prepared from embryonic human telencephalon tissues are able to express constitutively mRNA transcripts for cytokines and chemokines and treatment with pro-inflammatory stimuli as lipopolysaccharide or Aβ peptide led to increased expression of mRNA levels of these inflammatory molecules [14].

The role of inflammation is further emphasized by a number of clinical studies demonstrating that the long-term use of non-steroidal anti-inflammatory drugs may protect against AD. There are now a lot of published observational studies demonstrating that people who are known to be taking anti-inflammatory drugs considerably reduce their odds of developing AD and population studies have confirmed this negative association [15].

However, alternative hypothesises have been proposed. In particular, this effect has been suggested largely due to these drugs ability to inhibit angiogenesis. In fact, the brain endothelium secretes the precursor substrate for the beta-amyloid plaque and a neurotoxic peptide that selectively kills cortical neurons. So, antiangiogenic drugs targeting the abnormal brain endothelial cell might be able to prevent and treat this disease [16].

The long-term prospective association between dementia and the well known inflammation marker high-sensitivity C-reactive protein was evaluated in a cohort of Japanese American men who were seen in the second examination of the Honolulu Heart Program (1968-1970) and subsequently were re-examined 25 years later for dementia in the Honolulu-Asia Aging Study (1991-1996). In a random subsample of 1,050 Honolulu-Asia Aging Study cases and noncases, high-sensitivity C-reactive protein concentrations were measured from serum taken at the second examination; dementia was assessed in a clinical examination that included neuroimaging and neuropsychological testing and was evaluated using international criteria. Compared with men in the lowest quartile (<0.34mg/L) of high-sensitivity C-reactive protein, men in the upper three quartiles had a 3-fold significantly increased risk for all dementias combined, Alzheimer's disease, and vascular dementia. These data support the view that inflammatory markers may reflect not only peripheral disease, but also cerebral disease mechanisms related to dementia, and that these processes are measurable long before clinical symptoms appear [17].

On the other hand, several other investigations have shown increased blood levels of some cytokines, such as IL-1β and IL-6, and acute phase proteins (α-1-antichymotrypsin, ACT) in patients with clinical AD [18-21]. Therefore, altered immune responses in the brain and the peripheral blood appeared to be associated with the disease. Finally, plasma levels of ACT also correlated with the degree of cognitive impairment in AD patients form a case-control study [96] suggesting that peripheral markers of inflammation or impaired immune responses could be used for monitoring the progression of the disease.

Moreover, elevated levels of IL-6 in both brain homogenates and peripheral blood from AD patients have been reported [22]. These findings suggested that an important, but still largely unknown, interplay between brain and peripheral immune responses existed in the diseases.

In conclusion, the brain lesions associated with AD, which are referred to as neurofibrillary tangles and senile plaques, are characterized by the presence of a broad spectrum of inflammatory mediators, produced by resident brain cells, including neurons. Although secondary to the fundamental pathology caused by the presence of tangles and plaques, there is strong evidence that inflammation exacerbates the neuronal loss. Accordingly, several reports have appeared indicating that the risk of AD is substantially influenced by several polymorphisms in the promoter region, and other untranslated regions, of genes encoding inflammatory mediators. Alleles that favour increased expression of the inflammatory mediators or alleles that favour decreased expression of anti-inflammatory mediators are more frequent in patients with AD than in controls. The polymorphisms are fairly common in the general population, so there is a strong likelihood that any given individual will inherit one or more of the high-risk alleles [21].

## Results

A summary of data derivation from the “Conselice” investigation at the beginning of the study and after the five year follow up is reported in Table 1.

A list of variable investigated and their functional definition used in this study is reported in Tables 2 and 3.

**Table 1:** Description of population investigated at the beginning (1999/2000) and at the end of the follow up (2003/2004)

1999/2000					
Eligible	Non participants^1^	Final population	Prevalent AD	Cognitively NC ^2^	AD free
N= 1353	n = 337	n = 1016	n = 60	n = 19	n = 937

Followup2003/2004					

Reassessed population	Non reassessed ^3^	Final population	Incident ADdementia	Cognitively NC non classified	AD free cohort
N = 937	n = 133	n = 804	n = 109	n = 4	n = 695

^1^ Refusals n = 271; Deceased n = 59; Not found n = 7.

^2^ NC = non classified

^3^ Refusals n =74; Deceased n = 28; Not Found n = 31

**Table 2:** Genetic variables used in the connectivity map

Genetic variable (gene polymorphism)		
gene	SNP	Allele mutated
ACT	**rs 1884082**	**T**
APOE	**variation ε 2,3,4**	**ε 4**
HMGCR	**rs3761740**	**A**
IL-1 beta	**rs16944**	**T**
IL-6	**rs1800795**	**C**

**Table 3:** Phenotypic and clinical variables used in the connectivity map.

Variable	level
high ACT	> 400 ug/ml
high cholesterol	> 200 mg/dl
high CRP	> 0.3 mg/ml
high HDL	> 65 mg/dl
low HDL	< 40 mg/dl
high IL-6	> 5 pg/ml
high TNFa	> 20 pg/ml
high triglicerids	> 175 mg/dl
high Hcy	> 17 umol/l
high folate	> 5.3 mg/dl
high TSH	>0,4 mU/L
high vitB12	> 341 pg/ml
high BMI	> 28
Hypertension	> 140 mm hg
type II diabetes	positive

**Figure 1:**
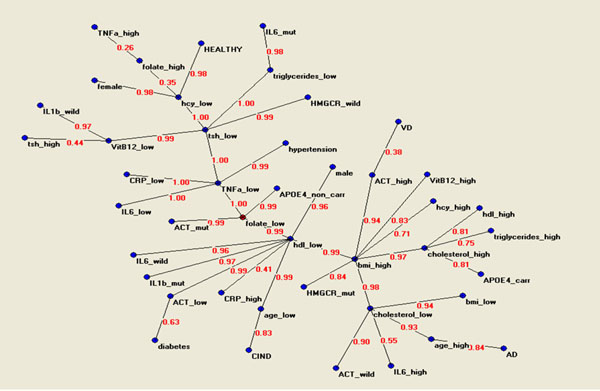
Connectivity map of 42 epidemiological, genetic and clinical variables showing different output such as prevalent AD, prevalent VD, prevalent CIND and control cases

The connectivity map related to 42 variables from the Conselice study data base focused upon the AD, VD and CIND prevalent cases during the follow up interval is shown in Figure 1. The map depicts the most relevant associations present in the data base. The figures on the connections lines are proportional to the strength of connections. Chronological age was the closest variable to prevalent AD. However, several major biological hubs were identified: 1) low blood cholesterol, 2) high BMI index, 3) low blood HDL, 4) low blood folate.

Different genotypic, phenotypic, clinical, pharmacological or habit variables converged to these diverse hubs or cluster of connectivity. Low blood cholesterol levels was the first hub directly linked with age. Elevated IL-6 blood levels and ACT geneotype appeared to influence low cholesterol levels. The second hub was represented by high BMI index; several other variables were connected on high BMI. Increased blood cholesterol, APOE 4 allele, increased blood hcy, increased ACT and VitB12, and the mutate allele of HMGCR gene. Low blood HDL was the third hub and several variable were linked to this hub such as, male gender, increased blood CRP levels, the mutated allele of IL-1 beta gene. The fourth hub was low blood folate linked to APOE 3 and 2 alleles and the mutated ACT allele.

Third and fourth hubs in the connectivity map were shared by prevalent CIND and VD cases. Low age was directly connected with the CIND clinical state. Whereas, increased blood ACT levels were directly linked with prevalent VD.

Cognitive healthy status at the end of the follow up was on the other extremity of the connectivity map; far away from CIND, VD and on the opposite site of AD.

## Discussion and conclusions

AD is a complex and multi-factorial disease, therefore, it is unlikely that a single biomarker may be determinant in the diagnosis or monitoring the progression of the disease.

The statistical analysis applied to elaborate biological and clinical data was a new enter in the field of biology and medicine. In fact, most common algorithms used in medicine are limited by the following limitations: 1) the analyses usually do not preserve the geometrical structure between variables when non linear relationships among variables are not evident. 2) another factor of uncertainty is how to establish precise associations between variables without predefined contiguity.

Here we used a new paradigm aimed to map variables and search for connectivity. In this analysis non linear association were preserved, explicit connection schemes were investigated and the complexity of dynamic interactions were preserved. The mathematics and philosophy of this analysis has been described in detail elsewhere [23]. Some application of similar kind of this analysis has already been focused to AD investigations with interesting findings [24].

Findings described here generated a connectivity map among variables and illustrated a rational path of biological variables leading to prevalent dementia.

Data presented here suggest that age, low cholesterol, high BMI, low HDL and low folate are major variable associated with the risk of AD, VD and CIND. CIND, as expected, were associated with a lower age at onset.

Our findings showed four major connecting nodes from the Conselice data base; these hubs linked apparently different factors to cognitive impairment and dementia via cholesterol, cholesterol gene dependent pathway, BMI and age. A new association among different immunological factors and lipid metabolism with incident dementia has also emerged.

In conclusion the connectivity map presented here on prevalent dementia extents previous observations from case/control investigations and population investigations and confirm that some immune factors indeed play a role in the pathogenesis of age-associated dementia by modifying metabolic and lipid variables and also show a new link between immunity, cholesterol metabolism and age related cognitive deterioration.

## Material and methods

### Data base generation

Data were collected from the elderly (65 year old or older) living in Conselice county in Northern Italy. Participants were interviewed, medically examined and cognitively evaluated in 1999. A blood sample from each subject was taken and each participant was given a computerized scan radiogram of the brain. After five years subjects underwent medical and cognitive re-evaluation. 937 elderly completed the follow up. A detailed description of the clinical protocol and the assessed variable has been already described elsewhere [25,26].

Diagnosis of dementia was performed according criteria of DSM-IV (1994). Clinical AD was defined using the NINCDS-ADRDA criteria [27]. Vascular dementia (VD) was diagnosed using NINDS-AIREN criteria [28].

Diagnosis of CIND was performed according methods already described [29].

### Statistical analysis

Conselice data base has the aim of increasing our understanding of the pathogenetic pathway leading to cognitive decline and dementia. This goal has been achieved through a new mathematical approach able to point out the relative relevance of each variable in representing major biological hub or aggregation point. This new paradigm of variables processing aims to create a semantic connectivity map in which: a) non linear associations are preserved, b) there are explicit connections schemes, c) the complex dynamics of adaptive interactions is captured. This method is based on an artificial adaptive system able to define the strength of the associations of each variable with all the others in any dataset, named the Auto Contractive Map (AutoCM). The architecture and the mathematic of AutoCM was invented, tested and implemented in C language, as described elsewhere [24]. The philosophy behind this approach is to pick up affinities among variables related to their dynamical interaction rather than to their simple contingent spatial position. This approach is suggested more suitable to describe a context typical of living systems in which there is a continuous complex change in the variables values among time. After the training phase, the weights matrix of the AutoCM represents the warped landscape of the dataset. We apply a simple filter (minimum spanning tree by Kruskal) to the weights matrix of AutoCM system to show the map of main connections between and among variables and the principal hubs of the system.

## Authors’ contribution

EG and MB created and developed the Auto Contractive Map; EP and IC performed laboratory analysis and genotyping; PF and GR collected samples and conceived of the Conselice Study of Brain Ageing; FL coordinated the application of statistical analysis of Conselice data base and contributed to design the clinical, epidemiological and genetic study.

## Competing interests

The authors declare that they have no competing interests.

## Acknownledgments

Research supported by Italian Ministry for Research and University, Pallotti Funds, Fondazione Cassa di Risparmio di Bologna e Fondazione del Monte di Bologna e Ravenna, Italy.

This article has been published as part of *Immunity & Ageing* Volume 7 Supplement 1, 2010: Predictive medicine, new diagnostic tools and prevention of diseases. The full contents of the supplement are available online at http://www.immunityageing.com/content/7/S1

## Abbreviations

ACT: alpha-1 antichymotrypsin; APOE: Apolipoprotein E; HMGCR: hydrossil-methyl-glutayl CoA reductase; IL-1 b: Interlukin-1 beta; IL-6: Interleukin-6; TNFa: tumor necrosis factor alpha; AD: patients with Alzheimer’s disease; CIND: patient with clinical diagnosis of cognitive impairment but no dementia; VD: patients with vascular dementia; Hcy: homocystein; Tsh: thyroid stimulating hormone; BMI: Body max index; HDL: high density lipoprotein
